# Screening for aneurysms of the abdominal aorta using a simple screening device

**DOI:** 10.1186/s13089-020-00192-5

**Published:** 2020-11-11

**Authors:** J. W. Brakel, T. A. Berendsen, P. M. C. Callenbach, J. van der Burgh, R. J. Hissink, M. van den Berg

**Affiliations:** Department of Surgery, Treant Zorggroep, Boermarkeweg 60, 7824 AA Emmen, Netherlands

**Keywords:** Abdominal aneurysm, Screening, Aorta Scan, Vascular surgery

## Abstract

**Introduction:**

Several countries advocate screening for aneurysms of the abdominal aorta (AAA) in selected patients. In the Netherlands, routine screening is currently under review by the National Health Council. In any screening programme, cost-efficiency and accuracy are key. In this study, we evaluate the Aorta Scan (Verathon, Amsterdam, Netherlands), a cost-effective and easy-to-use screening device based on bladder scan technology, which enables untrained personnel to screen for AAA.

**Methods:**

We subjected 117 patients to an Aorta Scan and compared the results to the gold standard (abdominal ultrasound). We used statistical analysis to determine sensitivity and specificity of the Aorta Scan, as well as the positive and negative predictive values, accuracy, and inter-test agreement (Kappa).

**Results:**

Sensitivity and specificity were 0.86 and 0.98, respectively. Positive predictive value was 0.98 and negative predictive value was 0.88. Accuracy was determined at 0.92 and the Kappa value was 0.85. When waist–hip circumferences (WHC) of > 115 cm were excluded, sensitivity raised to 0.96, specificity stayed 0.98, positive and negative predictive value were 0.98 and 0.96, respectively, accuracy to 0.97, and Kappa to 0.94.

**Conclusion:**

Herein, we show that the Aorta Scan is a cost-effective and very accurate screening tool, especially in patients with WHC below 115 cm, which makes it a suitable candidate for implementation into clinical practice, specifically in the setting of screening selected populations for the presence of AAA.

## Introduction

Aneurysms of the abdominal aorta (AAAs) occur in 2–5% of males aged > 65 years with a history of vascular disease or smoking [1]. However, the incidence of aneurysms of the abdominal aorta in patients with peripheral artery disease is higher (7.3–15%) [2].

When left untreated, AAAs are prone to keep growing and weakening the arterial wall, which may eventually result in aortic dissection or rupture, which carries a mortality rate of 50–80% [3]. In the Netherlands, approximately 700 patients die annually due to the consequences of an AAA, which includes aortic rupture as well as perioperative mortality [4]. It has been shown that AAA screening in men aged 65–75 years who smoke can significantly reduce mortality [5]. As a result, the Dutch National Health Council has recently commenced an investigation into the merits of a national screening programme for AAA [6]. To date, many countries are in various stages of implementing screening programmes for AAA, and in several countries without a national screening programme, screening is carried out by individual hospitals [7–10]. Typically, screening will be singular (once per patient), and directed at persons at risk: males between 65 and 75 years old who smoke or have a form of atherosclerosis (peripheral artery disease or coronary artery disease) or a positive family history of AAA. Whereas screening has the obvious benefit of detection and early treatment, there are also a number of disadvantages. First, not everyone diagnosed with AAA will ultimately succumb to it, which would mean that a screening programme could lead to considerable overtreatment. Darling et al. have shown that up to 10% of post-mortem diagnostics yielded an AAA while the cause of death was unrelated [10]. Although this is compensated by an upper limit to the screening age, a significant portion of the aneurysms detected through screening may never become clinically relevant—especially in the case of small aneurysms (< 4 cm in diameter). In addition, there are added costs of treatment and the costs of screening itself. Contrarily, early detection of (small) aneurysms may not only lower mortality rates, but also reduce cost and disease-related morbidity as, due to early management, costly interventions for advanced aneurysms may be averted. Moreover, screening for aneurysms can trigger treatment for other types of vascular disease, such as hypertension and hypercholesterolaemia as well as to initiate cardiovascular risk management. This reduces the likelihood of progressive vascular disease in these patients, which could also significantly reduce overall cost. The costs vs. benefits of screening are quite difficult to estimate, especially since different countries employ different financial systems to manage health care. However, reducing screening costs seems a priority because it is a major factor in the decision whether or not to implement a screening programme. Usually, screening for AAA would consist of an abdominal ultrasound and an outpatient visit to inform the patient of the result. Total costs per patient are estimated at $500 in the USA and average € 222 in the Netherlands, online research conducted by our department. This difference is mainly caused by higher costs of the ultrasound examination in the USA.

However, if the Aorta Scan is integrated into the outpatient visit, total costs per patient can be reduced to € 123 and when integrated in the general practice a further reduction of costs can be achieved till € 70 per patient. The purchase value of the Aorta Scan is just 10% of the ultrasound machine.

We propose here that the implementation of handheld screening devices that are cheap and easy to use can significantly reduce the costs, and thereby benefit the efficiency of AAA screening. Moreover, as handheld devices can also be used in primary care facilities, the scope of screening can be expanded by the implementation of such devices. To facilitate a screening programme in our own vascular surgery clinic, we evaluated the Aorta Scan BVI 9600. This device, which is basically an automated bladder scan device with added capability to measure the abdominal aorta, is easy to use, standardized, and certified for AAA measurement. The exam can be performed in minutes by relatively untrained personnel. We hypothesized that if the specificity, sensitivity and overall accuracy of this device are acceptable, it could be a valuable tool in any AAA screening programme. In addition, we were curious to analyse whether the implementation of this screening tool would lead to an increase in the incidence of AAA within our patient population.

## Methods

To test our hypothesis, we recruited 117 patients from our vascular surgery clinic (Treant Hospital, Hospital location Scheper, the Netherlands) between May 2014 and May 2017, and obtained written informed consent. Baseline characteristics are shown in Table 1. Patients that either had been diagnosed previously with AAA and were in follow-up and patients with peripheral arterial disease (PAD) were selected. Patients who underwent previous AAA repair were excluded, as well as patients unwilling or unable to comply with the study protocol. Through the inclusion process, we tried to include a ratio of 50/50 between patients with a known AAA and patients with PAD only (undiagnosed for AAA). All patients underwent first conventional abdominal ultrasound (measuring abdominal aorta diameter in the sagittal plane) in our radiology department, conducted by a single certified radiologist, who was blinded to any information related to the patient. The medium time needed to perform the ultrasound amounts to 15 min. Directly afterwards the patients underwent a 4-point measurement in the abdomen with the Aorta Scan in our vascular out-clinic department, by a health practitioner with less experience in ultrasound, see pictures below.

**Table 1:** Baseline characteristics of included patients (*N* = 117)

	Total group (*n* = 117)	Ultrasound < 3 (*n* = 59)	Ultrasound > 3 (*n* = 59)	*p*-value
Sex *n* (%)				*< 0.001*
Male	88 (75.2)	36 (61.0)	52 (89.7)	
Female	29 (24.8)	23 (39.0)	6 (10.3)	
Mean age in years (SD)	70.5 (8.5)	67.7 (8.4)	73.3 (7.7)	*< 0.001*
BMI *n* (%)				0.563
20–24	29 (24.8)	15 (25.4)	14 (24.1)	
25–30	69 (59.0)	33 (55.9)	36 (62.1)	
31–40	18 (15.4)	11 (18.6)	7 (12.1)	
> 40	1 (0.9)	0 (0.0)	1 (1.7)	
WHC in cm (SD)	103.0 (11.4)	101.6 (12.0)	104.4 (10.7)	0.184
Hypertension *n* (%)	70 (59.8)	33 (55.9)	37 (63.8)	0.386
Diabetes *n* (%)	32 (27.4)	18 (30.5)	14 (24.1)	0.440
Hypercholesterolaemia *n* (%)	31 (26.5)	17 (28.8)	14 (24.1)	0.567
History of smoking *n* (%)	112 (95.7)	55 (93.2)	57 (98.3)	0.176
Coronary heart disease *n* (%)	52 (44.4)	30 (50.8)	22 (37.9)	0.160


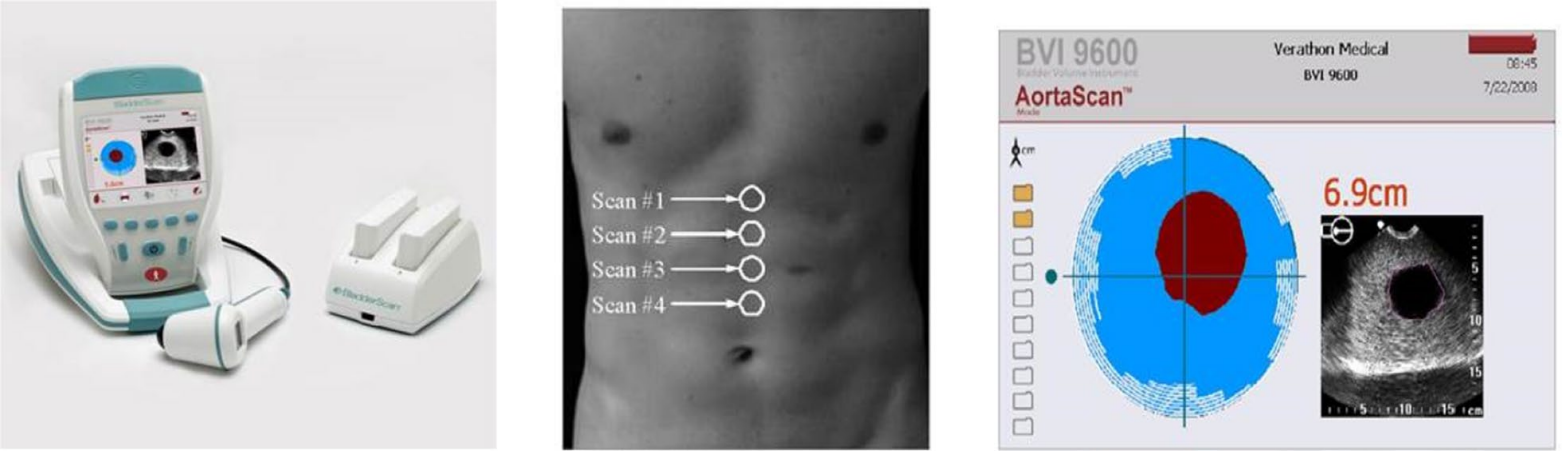
The medium time needed to perform the Aorta Scan amounts to 5 min and the widest diameter was taken.

The results of both methods were compared, and sensitivity, specificity, predictive values, inter-test agreement (kappa), and accuracy of the Aorta Scan were calculated using SPSS 23.0 (Table 2). Furthermore, we tested whether baseline characteristics like body mass index (BMI) and waist–hip circumference (WHC) affected the accuracy of the Aorta Scan. For this, the patients were divided in two groups, one group in which the results of the ultrasound and Aorta Scan corresponded and in which they did not correspond. A *t*-test was performed between these two groups for BMI and WHC.

**Table 2:** Methods to calculate sensitivity, specificity, PPV, NPV and accuracy

	Abdominal ultrasound		
	Positive	Negative	
Aorta Scan			
Positive	TP	FP	PPV = TP/(TP + FP)
Negative	FN	TN	NPV = TN/(FN + TN)
	Sensitivity = TP/(TP + FN)	Specificity = TN/(FP + TN)	Accuracy = (TP + TN)/All

*FN* false negative, *FP* false positive, *NPV* negative predictive value, *PPV* positive predictive value, *TN* true negative, *TP* true positive

## Results

Of the 117 patients included, 58 were positive and 59 were negative for AAA on conventional ultrasound (Table 3). Patients with AAA were significantly more often male and were older in age (Table 1). Of the 117 patients, 49 were already known with AAA, leading to an incidence (percentage of new AAAs) in our population of 9 patients (13.2%).

**Table 3:** Results of Aorta Scan compared to abdominal ultrasound

	Abdominal ultrasound		Total
	> 3	< 3	
Aorta Scan			
> 3	50	1	51
< 3	8	58	66
Total	58	59	117

**Fig. 1:**
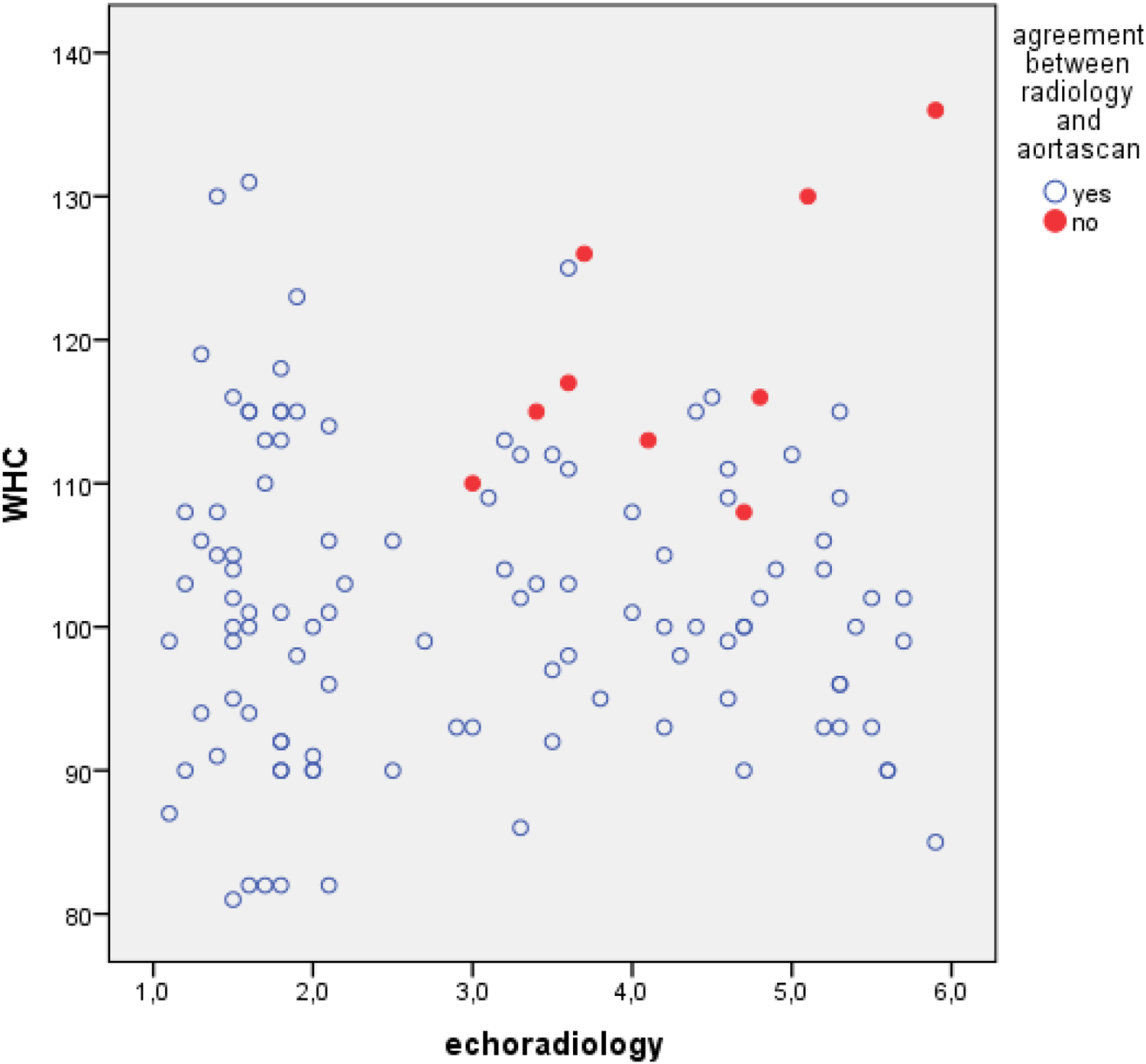
Plot between the WHC and measurement of AAA. Patients for whom disagreement was observed between Aorta Scan and conventional radiology are shown in red (*n* = 9)

When we review the Aorta Scan results, 51 patients were positive for AAA and 66 negative, resulting in a sensitivity of 50/58 = 0.86 and a specificity of 58/59 = 0.98 (Table 3). Positive predictive value was 50/51 = 0.98 for the Aorta Scan, while the negative predictive value was 58/66 = 0.88. The accuracy was determined at (50 + 58)/117 = 0.92. Inter-test agreement (Kappa value) was 0.85. We proceeded to look at individual parameters that we hypothesized might affect accuracy of the Aorta Scan, such as BMI and WHC. It was observed that the WHC was significantly higher in the group in which the echo and Aorta Scan did not correspond (117 vs 101, *p* < 0.001), as was the BMI (31 vs 27, *p* = 0.007). Notably, when we plotted the WHC against measurement of the AAA, we found that high WHC corresponded with disagreement between the two modalities, leading us to hypothesize that high WHC might cause erratic values for the Aorta Scan (Fig. 1). When a cut-off WHC of 115 was applied to the data, the sensitivity raised to 46/48 = 0.96 and the accuracy to (46 + 47)/96 = 0.97, with a corresponding Kappa value of 0.94 (Table 4). BMI or BMI/WHC combined had no significant influence on accuracy (data not shown).

**Table 4:** Results of Aorta Scan compared to abdominal ultrasound excluding patients with waist–hip circumference > 115 cm

	Abdominal ultrasound		Total
	> 3	< 3	
Aorta Scan			
> 3	46	1	47
< 3	2	47	49
Total	48	48	96

## Discussion

As research data into the pathological behaviour of aneurysms of the abdominal aorta continue to be gathered, more countries implement screening programmes to test predisposed patients for the presence of AAA. Patients with AAA were significantly more often male and were older in age (Table 1). This is in line with previous literature [11], showing that our study population is representative for the AAA population.

Screening usually involves ultrasonography, which is a costly and complex investigation that is generally impossible to perform in a primary care facility or a family practice. In addition, several countries have now implemented cardiovascular risk management in primary care facilities which means that screening for AAA could be performed in a similar setting. The introduction of a simple handheld screening device would enable AAA screening to be moved from vascular clinics and general hospitals towards primary care facilities and outpatient clinics, quite similar to the screening, diagnostics and treatment of diabetes by family practitioners and specialized nurses [12]. Such developments would benefit health care cost-effectiveness and would promote more patients to participate in screening for a host of pathologies, including AAA. While screening always comes at great financial expense, costly treatment and morbidity may be spared through the early detection of AAA.

In this study, we tested a handheld aortic scanning device and determined its accuracy in detecting the presence and absence of AAA. We confirmed that the accuracy of this device is sufficient to be used as a screening tool for the general population at risk, in particular those patients with a WHC < 115 cm. In such cases, we would recommend using the Aorta Scan or a similarly certified device to screen for AAA.

Nine patients who were diagnosed with AAA during our study visited our out-clinic department for peripheral artery disease, and would in daily practice not have been subjected to examination of their aorta. Their AAA would have remained undiagnosed if they had not participated in the study. A screening programme for AAA in predisposed patients as suggested by the Dutch National Health Council should be considered. The incidence of 13.2% is high compared to the general population (which has an AAA incidence of approximately 2%), but comparable to the incidence of AAA in patients with peripheral artery disease (7.3–15%) [2].

As we wanted to study the effectiveness of the Aorta Scan in diagnosing true-positive cases (people with an actual AAA as proven by ultrasound), we selected patients from our outpatient clinic with a known history of AAA up to a percentage of 50% of our study population. While this creates a selection bias (the study group does not reflect the general population), the advantage is that we included the largest number of AAAs in any study known to date. Because we implemented such a high incidence of AAA (58 out of 117), we were able to very accurately study the results in the rare case of an AAA, and determine the outcome parameters within statistical significance. If a standard screening group was used, with a normal incidence of AAA of between 2 and 2.5%, only 2 or 3 patients in our group would have been diagnosed with AAA, which would have compromised our ability to reach significance without greatly increasing the study population. The accuracy of the Aorta Scan was tested by other researchers as well, such as Nguyen et al., however because they had such a low incidence of AAA, their results might be easily disturbed by a false result in the Aorta Scan (for instance, a negative result by accident regardless of the test subject) [13]. Kappa measurement corrects for precisely such chance results. Furthermore, Nguyen mentioned that there were several unspecified technical problems with the device which need improvement [13]. We used the same device in our study, but we did not investigate technical problems.

As alternative to using a cheaper device such as the Aorta Scan, it has been suggested by others that novice trainees might be as good at detecting AAAs as an experienced ultrasonographer and thereby reduce costs as well (salary) [14]. However, this solution would not extend the amount of patients that could be screened in primary practices. In addition, others have shown as well that a small sized ultrasound paired with relatively short training in about 2 h enables effective screening for AAA in a clinical setting [15, 16].

In our study, the exam was performed by a single certified radiologist and the accuracy results are probably representing a higher performance obtainable. The results with the Aorta Scan can be less accurate when performed by health practitioner with less experience [17].

In summary, these data confirm and strengthen existing data that the Aorta Scan BVI 9600 is an easy, cost-effective, and reliable tool for measuring the aortic diameter, especially in patients with WHC ≤ 115 cm. We support the opinion that screening for AAA is important in selected patient populations, and we suggest the use of a handheld device such as the Aorta Scan, as it cuts costs and time, while preserving accuracy and precision of AAA diagnostics. Furthermore, new cases of AAA can be identified earlier and treated, which we believe is advantageous to the health care we continue to provide.

## Conclusion

We showed that the Aorta Scan is a cost-effective and very accurate screening tool, especially in patients with WHC below 115 cm, which makes it a suitable candidate for implementation into clinical practice, specifically in the setting of screening selected populations for the presence of AAA.

## Data Availability

An abstract of the dataset generated during and analysed during the study can be found in Tables 1, 2 in this manuscript. All datasets used and analysed during the study are available from the corresponding author on request.

## Abbreviations

AAA: Aneurysms of the abdominal aorta
PAD: Peripheral arterial disease
BMI: Body mass index
WHC: Waist–hip circumference
FN: False negative
FP: False positive
NPV: Negative predictive value
PPV: Positive predictive value
TN: True negative
TP: True positive
